# Effectiveness of clozapine in treatment-resistant schizophrenia

**DOI:** 10.4103/0019-5545.55955

**Published:** 2005

**Authors:** Janakiraman Raguraman, K. John Vijay Sagar, R. Chandrasekaran

**Affiliations:** * Senior House Officer Department of Psychiatry, Doncaster Royal Infirmary, Sheffield Care Trust, DN2 5LT, UK; ** Assistant Professor Department of Psychiatry, Tirupati Medical College, Andhra Pradesh, India; *** Professor and Head Department of Psychiatry, JIPMER, Pondicherry 605006

**Keywords:** Treatment-resistant schizophrenia, psychopathology, clozapine

## Abstract

**Background:**

Clozapine has been shown to be superior to chlorpromazine in improving the positive and negative symptoms of schizophrenia. However, technical experience with clozapine in Indian patients has not been documented.

**Aim:**

To assess the improvement in psychopathology of treatment-resistant schizophrenia with clozapine therapy and to study the relationship between sociodemographic and various psychopathology variables among patients with treatment-resistant schizophrenia.

**Methods:**

Twenty-two patients with treatment-resistant schizophrenia were evaluated using the Positive and Negative Syndrome Scale (PANSS) for schizophrenia, Calgary Depression Scale, Global Assessment of Functioning (GAF) Scale and Abnormal Involuntary Movement Scale (AIMS). These scales were used to determine the level of psychopathology, depression, overall functioning and severity of abnormal involuntary movements in the patients. The patients were admitted to the hospital for a short time to initiate clozapine therapy. At discharge, patients were stabilized on 300–400 mg/day of clozapine. The patients were re-evaluated after 20 months.

**Results:**

The study group showed better global functioning after clozapine therapy. The therapy was well-tolerated though moderate side-effects were seen. Suicidal thoughts declined with clozapine therapy. There was a significant reduction in the negative symptom and general psychopathology scores of PANSS.

**Conclusion:**

Clozapine has therapeutic efficacy in some but not all treatment-resistant patients with schizophrenia.

## INTRODUCTION

Since the discovery of clozapine in 1958, in Berne, Switzerland and its introduction in 1989, it was demonstrated to be superior to chlorpromazine in improving the positive and negative symptoms of schizophrenia.^1^ In this study on 267 patients with schizophrenia who met strict criteria for treatment resistance, 30% of clozapine-treated patients improved after 6 weeks in comparison with 4% of those receiving chlorpromazine.

The introduction of clozapine into clinical practice helped to set the stage for a variety of new perspectives on antipsychotic drug treatment, development and outcome assessment. Clozapine is the first drug to demonstrate a qualitative difference in the propensity to produce neuroleptic side-effects.^2^ Treatment-resistant schizophrenia is a common problem in patients with schizophrenia; it creates a huge economic burden for society.

A patient with schizophrenia who does not respond to at least two adequate trials of classical antipsychotics should be classified as being treatment-resistant. Although the term treatment-resistant schizophrenia proposed by Kane *et al.*^1^ has been widely used, the definition of adequate trials tends to be less rigorous recently.

According to the review by Conley and Buchanan,^3^ a 4–6-week (rather than a strict 6-week) trial of a classical antipsychotic equivalent to 400–600 mg/day of chlor-promazine should be regarded as the standard for an adequate trial.

Since the publication of the landmark study by Kane *et al.*^1^ clozapine has been regarded as an effective treatment for patients with treatment-resistant schizophrenia. The benefit of clozapine has been supported by evidence of short-, medium- and long-term treatment.^1^,^4^–^6^

Baseline predictors of response to clozapine such as male sex, subtype paranoid schizophrenia, older age at onset of illness, shorter duration of illness, higher level of pre-treatment and acute extrapyramidal symptoms (EPS) have shown a good therapeutic response.^7^

Rosenbeck *et al.*^8^ found that patients with a higher level of baseline symptoms had a greater reduction in symptoms and greater improvement in the quality of life at 12 months of treatment. Schizophrenia is the most common primary diagnosis in suicide enquiry cases. A study conducted by Duggan *et al.*^9^ showed that an average of 53 suicides could have been avoided each year by prescribing clozapine to these patients, considering their compliance suitability. Clozapine should be considered in the treatment of both neuroleptic-resistant and neuroleptic-responsive patients with schizophrenia who have persistent suicidal thoughts or behaviour.^10^

Open clinical trials conducted in the early 1970s suggest that clozapine is an effective antipsychotic drug that lacks extrapyramidal side-effects. Clozapine was found to be superior in 79% of the controlled trials that compared it with another antipsychotic drug.

In a study by Lieberman *et al.*^7^ 50% of treatment-resistant patients responded effectively to clozapine. Spirak showed an improvement in tardive dyskinesia, akathesia and parkinsonism.

A recent meta-analysis^11^ reviewed 2560 randomized participants of 31, mainly short-term, trials. Clozapine was clearly more effective than conventional antipsychotics in reducing symptoms both in treatment-resistant and non-resistant patients. No convincing evidence was found that the superior clinical effects of clozapine results in an improved level of functioning.

Technical experience with clozapine in Indian patients has not been documented. Assuming that clozapine shows a better outcome in treatment-resistant patients with schizophrenia with or without suicidal ideation and EPS, we undertook this study to assess the effectiveness of clozapine under a natural follow-up evaluation and to study the relationship between sociodemographic and various psychopathology variables in treatment-resistant schizophrenia.

## METHODS

The Department of Psychiatry at the Jawaharlal Institute of Postgraduate Medical Education and Research (JIPMER) conducts a weekly outpatient clinic for schizophrenia and related disorders. A large number of patients do not respond to adequate dosages of more than one class of antipsychotic drugs. As the administration decided to supply clozapine free of cost to the patients, a decision was taken to identify treatment-resistant patients with schizophrenia who may benefit from clozapine therapy.

During the year 2002, 35 patients were evaluated. Before evaluation, each patient was fully informed of the benefits, risks and potential side-effects of clozapine therapy, and signed informed consent was obtained. The patients underwent a comprehensive physical examination; blood cell counts, liver function tests, and blood glucose levels were tested, and ECG and EEG were recorded. Patients who suffered from liver dysfunction, epilepsy, diabetes and had a poor haematological profile were not considered for clozapine therapy. This resulted in the exclusion of 5 patients. The remaining 30 patients were further evaluated using the Positive and Negative Syndrome Scale (PANSS) for schizophrenia,^12^ Calgary Depression Scale,^13^ Global Assessment of Functioning (GAF) Scale^14^ and the Abnormal Involuntary Movement Scale.^15^,^16^

We determined the level of psychopathology, depression, overall functioning and severity of abnormal involuntary movements which, in our view, can be influenced by clozapine. The majority of patients were admitted to the hospital for a short time, to taper the drugs they were taking and to introduce clozapine in a gradual manner. At the time of discharge, the patients were stabilized on 300–400 mg/ day of clozapine. Of these 30 patients, 8 were not drug-compliant and did not report regularly for follow-up. Finally, 22 patients who were on regular follow-up and more drug compliant, a fact confirmed by their close relatives, were re-evaluated after an observation period of 20 months. Improvement was defined as a 25% reduction in the total PANSS score from the baseline.

Among the 22 patients included in this study, 14 (63.6%) were men and 8 (36.4%) were women. The mean age of the patients was 32.4 (±10.4) years. Fifteen (68.2%) were single and 7 (31.8%) were married. Fourteen were from urban and 8 from rural areas. Ten patients (45.5%) had a family history of psychiatric illness and 12 (54.5%) had no significant family history.

## RESULTS

Fifty per cent of the study group responded to clozapine therapy during the observation period of 20 months. Four patients (18.2%) had a paranoid schizophrenia subtype, while 18 (81.8%) had an undifferentiated subtype. Table 1 compares the psychopathology of the patients at baseline and at final assessment. Comparison of various parameters in improved and unimproved groups at baseline is given in Table 2.

**Table 1:** Comparison of psychopathology at baseline and on final assessment

Variable	Before clozapine	After clozapine	*t*
PANSS score	91.3 (17.7)	66.0 (19)	8.4*
Positive symptom score	13.2 (7.2)	11.1 (5)	1.3
Negative symptom score	31.6 (8.9)	22.6 (9.2)	6.6*
General Psychopathology Score	45.9 (11.1)	33.4 (10.8)	6.9*
Calgary Depression Score	4.8 (5.4)	3.7 (5.3)	1.2
Global Assessment of Functioning Score	30.4 (13.9)	50.0 (15.7)	−5.3*
Suicidal Ideation Score	0.73 (1.0)	0.45 (0.9)	1.3
Abnormal Involuntary Movement Score	3.5 (7.5)	2.5 (5.6)	1.9
Abnormal Involuntary Movement Score	3.5 (7.5)	2.5 (5.6)	1.9

* p<0.000

**Table 2:** Comparison of baseline parameters between the improved and not improved groups

Variable	Improved (*n*=11)	Unimproved (*n*=11)	*t*
PANSS score	85.8 (20)	96.9 (13.8)	−1.5
Positive symptom score	12.6 (5.2)	13.8 (8.9)	−0.3
Negative symptom score	28.9 (9.9)	34.2 (7.3)	−1.4
General Psychopathology Score	42.4 (12)	49.3 (9.4)	−1.5
Calgary Depression Score	3.6 (5.1)	6 (5.6)	−1.0
Global Assessment of Functioning	27.2 (12.7)	33.6 (15)	−1.0
Suicidal Ideation Score	0.5 (0.8)	1.0 (1.1)	−1.2
Abnormal Involuntary Movement Score	1.8 (4.1)	5.2 (9.7)	−1.0
Total duration of illness (years)	8.2 (9.1)	6.5 (8.1)	0.4
Age onset of illness (years)	24.6 (7.1)	25.3 (8.6)	−0.2
Duration of clozapine therapy (months)	21.3 (17.8)	23.2 (16.2)	−0.2

## DISCUSSION

The results of this retrospective study are consistent with that of previous studies,^1^,^3^,^4^,^7^ which demonstrated that clozapine has therapeutic efficacy in some but not all patients with schizophrenia who are resistant to standard antipsychotic drugs.

Fifty per cent of the study group responded to clozapine therapy during the observation period of 20 months, which is consistent with the results of Lieberman *et al.*^7^ Other studies reported response rates of 30%-61%.^4^ Differences among these studies may be related to the length of trial, characteristics of the subjects and the responder's criteria used. The responder's criteria used in our study was a 25% reduction in the PANSS score from the baseline.

The study group showed a better global functioning after clozapine therapy. None of the patients showed deterioration in abnormal involuntary movements, rather, a marginal improvement was noticed in the majority of patients. In previous studies, such as the one by Lieberman *et al.*^7^, older age at onset, shorter duration of treatment and fewer psychiatric episodes were found to be associated with good response but they were not shown to be significant in our study.

There was no difference in response between men and women, unlike in other studies where men responded better.^7^ A significant improvement with clozapine was seen in the urban population, probably owing to good understanding of the illness, family support and better access to hospital. Patients with a family history of psychiatric illness did not show any significance with improved or not improved category. Patients with paranoid schizophrenia showed a better response compared to undifferentiated schizophrenia. This is consistent with the findings of Lieberman *et al.*^7^

Though the reduction in positive symptoms was favourable, it was not significant as in other studies.^7^

A significant reduction of negative symptom and general psychopathology symptom scores of PANSS was noted in our study despite similar improvement not being noted in depression. The Abnormal Involuntary Movement Scale (AIMS) scores suggest a possible direct effect of clozapine on primary negative symptoms.

Suicidal thoughts were found to decline with clozapine treatment as measured by the subscale of the Calgary Depression Scale. Clozapine was relatively well-tolerated, though moderate side-effects such as hypersalivation, drowsiness and anergia occurred. They generally abated within 10–12 weeks. Patients selected for the study were totally free of seizures and haematological side-effects.

Clozapine has brought new hope to many individuals with treatment-resistant schizophrenia. This new hope is not only because of the direct effects of medication itself but also because of the absence of most of the disabling side-effects of other neuroleptics.

The limitations of this study are a small sample size, absence of a control group and limited duration of follow-up. Future research should include a large sample with an appropriately matched control group and longer duration of study with periodic, regular follow-up.
